# The “Canopy Approach”: Case Series Using Immersive Virtual Reality for Bottom-Up Target-Based Preoperative Planning in Pediatric Neurosurgery

**DOI:** 10.1227/neuprac.0000000000000038

**Published:** 2023-04-14

**Authors:** Grace Y. Lai, Ryan R.L. Phelps, Nilika S. Singhal, Joseph E. Sullivan, Adam L. Numis, Kurtis I. Auguste

**Affiliations:** * Department of Neurological Surgery, University of California San Francisco, San Francisco, California, USA;; ‡ Department of Neurology, University of California San Francisco, San Francisco, California, USA

**Keywords:** Virtual reality, Preoperative planning, Pediatric neurosurgery, Pediatric epilepsy surgery, Pediatric brain tumor

## Abstract

**BACKGROUND:**

Virtual reality (VR) is increasingly used for trajectory planning in neurosurgery.

**OBJECTIVE:**

To describe a case series showing the application of immersive VR involving both “top-down” from skull to lesion and “bottom-up” from lesion to skull approaches for trajectory planning in pediatric neurosurgical patients.

**METHODS:**

We detail the preoperative and intraoperative application of VR and clinical courses of 5 children (aged 7-14 years) with anatomically challenging intraparenchymal lesions that posed operative risks to nearby vascular anatomy and fiber tracts. Preoperative planning consisted of standard presurgical evaluation with computed tomography and magnetic resonance imaging used to render 3-dimensional models that could be viewed and manipulated using desktop software and immersive VR headsets and hand controllers by the surgeon and family. Patient satisfaction was evaluated by survey. Surgical outcomes were degree of seizure control or extent of resection.

**RESULTS:**

Three patients underwent lesion resection and 2 laser ablation. Modifications to 2-dimensional and “top-down” VR trajectory plans were made after “bottom-up” navigation in all cases. All families reported that the VR enhanced their understanding of the procedure. There were no complications, and no patients suffered permanent neurological deficits postoperatively. Gross total resection was achieved in all lesional cases, and patients with epilepsy achieved seizure freedom at 2 years postoperatively.

**CONCLUSION:**

Immersive VR allows operative corridors to be virtually traveled and viewed from a “top-down” and “bottom-up” perspective, as if looking up from under a forest canopy of overlying anatomy, for optimal trajectory planning and improvement of family understanding in pediatric neurosurgery.

**ABBREVIATIONS:**
360°VR: 360° virtual reality
EEG: electroencephalogram
FCD: focal cortical dysplasia
PROCESS: Preferred Reporting Of CasE Series in Surgery
SRP: SuRgical Planner
SNAP: Surgical Navigation Advanced Platform
VR: virtual reality.

Virtual reality (VR) is increasingly used in many areas of neurosurgery to aid in surgical approach planning,^1-6^ training,^7-11^ and patient education.^2,12^ Transformation of 2-dimensional (2D) DICOM images into 3-dimensional (3D) projections in an immersive environment allows experience of patient-specific anatomy surrounding geometrically complex lesions. Integration of 3D projections with microscope and endoscope visualization allows for intraoperative augmented reality image guidance.^13-15^

As opposed to planning on a 2D stereotactic station, immersive VR offers multiple perspectives of the surgical lesion amid surrounding structures in a 3-dimensional environment that simulates the anatomy that one would encounter from the surgeon's view. Although interfacing with 2D and 3D images is traditionally performed on 2D interfaces, immersive headsets allow surgeons to temporarily occupy these 3D spaces when “immersed.” Furthermore, “fly-through” capabilities allow the surgeon to explore different trajectories from the skull down to the lesion while simultaneously visualizing traversing anatomy with the same vantage point.^2,11,16^ At our institution, we have employed the use of 360° immersive VR for patient education, preoperative planning, and intraoperative guidance. Over time, we established an algorithm for trajectory planning for an iterative trajectory improvement process: optimal trajectories are first planned using traditional 2D multimodal images and then from a “top-down” VR fly-through from the surgeon's perspective from a variety of planned entry points to the target lesion. Finally, we discovered that a second fly-through from *within* the brain at various anatomic boundaries of the lesion up through traversing structures to the skull, or a “bottom-up” approach, can help identify unique trajectories that may be missed with a top-down approach. This “bottom-up” perspective can be described as a *Canopy Approach*, as if peering up through a forest tree canopy. As opposed to moving across multiple surface locations peering down at a target to identify routes clear of traversing structures, one can more easily scan for “clear paths” from above, much like light peering through an overhanging tree canopy. We argue that this 3-step process can help improve trajectory planning across pediatric neurosurgical procedures including lesion resection, stereo-EEG placement (sEEG), and laser ablation.

We report 5 cases of pediatric patients with anatomically complex brain lesions for which the canopy approach with immersive VR was applied to study anatomic relationships and to optimize the operative approach.

## METHODS

Since 2017, our institution has used immersive VR for surgical planning, patient education, and intraoperative guidance in 237 pediatric patients with a variety of pathologies including epilepsy, tumors, and vascular malformations. Through this experience, we developed an algorithm for trajectory planning using the “Canopy Approach.” In this article, we describe 5 retrospective nonconsecutive cases from our center for which we intentionally applied the canopy approach for preoperative planning and discuss advantages of incorporating “bottom-up” planning. Demographic information, lesion type, and imaging modalities used for the creation of VR projections are listed in Table 1. Informed consent was obtained from each family, who consented to the publication of their child's images. The study was approved by the University of California, San Francisco Institutional Review Board. Follow-up was for a minimum of 2 years after the procedure described. Outcomes of interest for lesional cases were extent of resection based on the postoperative MRI and tumor recurrence. The outcome for interest of nonlesional epilepsy cases was seizure frequency postprocedure up to latest clinic follow-up. This case series has been reported in line with the Preferred Reporting Of CasE Series in Surgery (PROCESS) Guideline.^17^

**TABLE 1.** Demographic Characteristics, Clinical Characteristics, Imaging Modalities Used for VR) Planning, and Surgical Procedure for Each Case

Case number	Age, sex	Pathology	Preoperative imaging	Procedure (s)
1	14 y, male	Parasagittal synovial sarcoma of the brain	MRI T1 pre- and postcontrast, T2, FLAIR, and diffusion sequences magnetic resonance venogram	Left frontotemporoparietal craniotomy for resection of lesion
2	8 y, female	Intractable epilepsy with left posterior medial temporal neuroepithelial tumor of the young and fibrous dysplasia	MRI T1 pre- and postcontrast, T2, FLAIR, and diffusion sequences DTI	Left occipitofrontal craniotomy for resection of lesion and tumor focus
3	13 y, male	Intractable epilepsy with left parietal focal cortical dysplasia	MRI T1, T2, FLAIR, and diffusion sequencesDTI	Left parietal craniotomy with intraoperative electrocorticography for resection of lesion
4	11 y, male	Focal epilepsy and electrical status epilepticus in sleep with previously laser-ablated hypothalamic hamartoma	MRI T1 pre- and postcontrast, T2, FLAIR, and diffusion sequences	Bifrontal burr holes for laser ablation
5	7 y, male	Intractable focal epilepsy, nonlesional after resection of focal cortical dysplasia	MRI T1, T2, FLAIR, and diffusion sequencesDTICT angiogram	1. Stereo EEG electrode placement2. Left frontal burr hole for laser ablation

CT, computed tomography; DTI, Diffusion tensor imaging; EEG, electroencephalogram; FLAIR, fluid-attenuated inversion-recovery.

### 360°VR Model Construction

2D DICOM images from preoperative volumetric MRI and/or computed tomography scans (at most 1 mm in all planes) were reconstructed and fused to create a comprehensive 360° virtual reality (360°VR) model using proprietary software included in the SuRgical Planner (SRP; Surgical Theater). Each 360°VR model was volume-rendered such that all structures within a selected intensity range appear in 3D. Imaging sequences were fused in the same 3D space, allowing for any combination of scans to be shown at the same time. Thresholding and manual segmentation were performed to highlight anatomy such as the ventricles, blood vessels, and fiber tracks. Diffusion tensor imaging (DTI) was postprocessed using nordicBrainEx (NordicNeuroLab).

### Preoperative Planning

3D models created for VR evaluation were studied using the SRP 2D touchscreen desktop planning software and with immersive VR headsets and hand-held controllers (Oculus). The model can be continuously manipulated during fly-throughs using selective clipping and opacity control tools to visualize relevant structures because the user navigates along a trajectory. 3D markers were used to reference specific anatomy.

### The “Canopy Approach”

Virtual VR planning was first performed using a “top-down” approach from the skull to the target lesion through intervening cortical anatomy and vasculature. An optimal “top-down” trajectory was assigned to minimize disruption of critical structures. Navigation was then performed using a “bottom-up” approach from within the intracranial space at the starting point of the pathology through overlying cortical anatomy and vasculature. An optimal “bottom-up” corridor leading out to the cortical surface was assigned.

### Intraoperative Navigation

360VR models were imported from the SRP to the Surgical Navigation Advanced Platform (SNAP; Surgical Theater) and used for intraoperative navigation in conjunction with the StealthStation S7 neuronavigation system (Medtronic). SNAP's integration with the navigation software allowed automatic tracking of the navigation probe's position and orientation and adjusts the 360VR model accordingly. The 360VR model can also be adjusted manually based on the surgeon's preference.

## RESULTS

### Case 1

#### Presentation and Initial Management

**FIGURE 1.**
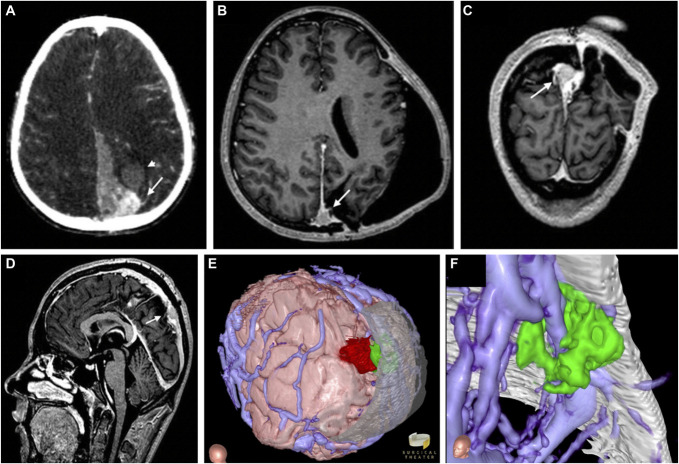
Hemorrhagic parietal synovial sarcoma. **A**, Axial noncontrast computed tomography shows a hyperdense lesion (arrow), intraparenchymal hematoma (arrowhead), interhemispheric hemorrhage, and 6 mm of midline shift. Postoperative **B**, Axial, **C**, Coronal, and **D**, Sagittal T1-weighted MR images show a residual contrast-enhancing lesion (arrows) along the superior sagittal sinus. **E**, Three-dimensional reconstruction of blended computed tomography and magnetic resonance imaging recreates the hemorrhagic tumor resected during the patient's first surgery (crimson) and residual tumor underlying the craniotomy edge (green). **F**, A “bottom-up,” intracranial vantage point reveals margins of the tumor underside of the superior sagittal sinus extending contralaterally.

A 14-year-old boy with a history of a large left parietal intraparenchymal hemorrhagic synovial sarcoma (Figure 1A) status after emergent decompressive hemicraniectomy and subtotal tumor resection is presented. Postoperative MRI (Figure 1B-1D) showed residual disease along the superior sagittal sinus, which seemed occluded and raised concern for sinus invasion. Given the pathological diagnosis, leaving behind residual tumor would negatively affect his oncologic prognosis and the family agreed to proceed with reresection.

### VR-Assisted Preoperative Workup

Critical evaluation of the patient's lesion by 2D MRI was difficult given its location along the parietal convexity. An initial plan based on both 2D rendering and a “top-down” immersive VR fly-through was to approach the lesion through the original craniectomy with minimal extension (Figure 1E). However, the intracranial “bottom-up” Canopy vantage point also revealed a rim of tumor expanding across midline (Figure 1F and Video 1). Thus, the plan was a larger extension of the original craniectomy to allow for access to both sides of sinus and lesion.

#### Postoperative Course

Surgery was uncomplicated, and the patient remained at neurological baseline. Gross total resection was achieved on postoperative imaging. He underwent adjuvant radiation later. There was no evidence of recurrent disease at a 4-year follow-up.

### Case 2

#### Presentation and Initial Management

**FIGURE 2.**
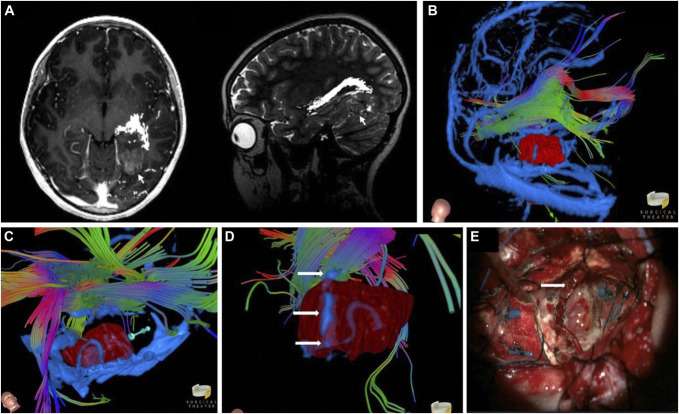
Left temporal neuroepithelial tumor of the young (PLNTY). **A**, Preoperative axial T1 and sagittal T2-weighted magnetic resonance **B**. Virtual reality 3-dimensional view from a posterior-lateral approach with the proposed operative trajectory to lesion (red). The optic radiations travel superiorly away from the lesion. **C**, “Bottom-up” view reveals a serpiginous vessel along the deepest surface of the lesion with the proposed posterior trajectory (turquoise arrow). **D**, High-magnification view from a lateral view shows a possible *en passage* vessel (arrows) traveling through the lesion and toward the optic radiations. The deep serpiginous vessel is seen in silhouette through a partially translucent lesion. **E**, Intraoperative image reveals a fully dissected *en passage* vessel (arrow) and the deep serpiginous vessel (arrowheads).

An 8-year-old girl with a 1-year history of intractable focal seizures with secondary generalization secondary to a well-circumscribed heterogeneous left posterior left temporal mass is presented (Figure 2A). Electroencephalogram (EEG) revealed focal discharges originating in the left occipital region, and the decision was made along with our interdisciplinary epilepsy board to proceed with surgical resection.

#### VR-Assisted Preoperative Workup and Intraoperative Findings

Based on 2D imaging, a subtemporal approach was initially considered. However, inspection of the 3D model favored a prone posterior approach to avoid the transverse-sigmoid junction and a shorter measured distance to the lesion from the cortical surface. 2D preoperative imaging did not show any distinct vasculature, but fly-through VR analysis revealed a series of vessels along the lateral tumor surface and suggested an *en passage* vessel (Figure 2B and 2C and Video 2). A “top-down” fly-through was used to identify an initial trajectory, which was further refined using the “bottom-up” Canopy view of the surrounding structures. In addition, the canopy approach identified a serpiginous vessel along the medial border of the lesion, which was used as a limit for extent of resection (Figure 2D, Video 2). The presence of the VR-depicted *en passage* vessel was encountered and preserved during surgery, and the medial serpiginous vessel was visualized demarcating the medial margin of resection (Figure 2E).

#### Postoperative Course

Surgery was uncomplicated. Postoperatively, the patient had a right superior quadrantanopsia, which resolved in 2 weeks. Imaging confirmed gross total resection. Pathological diagnosis was polymorphous low-grade neuroepithelial tumor of the young (World Health Organization grade I) and focal cortical dysplasia (FCD) type 3B. She remained seizure-free and without recurrent radiological disease on surveillance imaging at 4 years post-treatment.

### Case 3

#### Presentation and Initial Management

**FIGURE 3.**
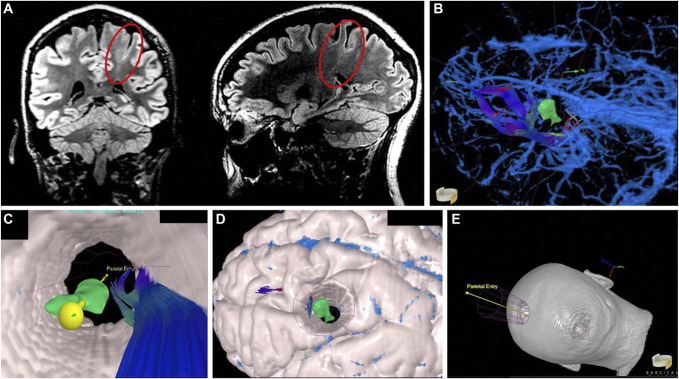
Left parietal focal cortical dysplasia. **A**, Coronal and sagittal fluid-attenuated inversion-recovery sequences reveal a hyperintense transcortical, “transmantle sign” (highlighted circle). **B**, Parietal entry point and **C**, “Canopy Approach” views of an optimal corridor to the lesion avoiding the neighboring motor tracts. **D**, Design of the potential transcortical approach using reconstructed parenchymal anatomy. **E**, Reconstructed skull and scalp planes used for planning optimal head positioning.

A 13-year-old boy with a 4-year history of intractable left focal seizures and concordant MRI fluid-attenuated inversion-recovery abnormality involving the deep and medial aspects of the left postcentral sulcus tracking to the ventricle (Figure 3A) is presented. Positron emission tomography (PET) imaging demonstrated hypometabolism in the corresponding region. EEG captured multiple seizures arising from the left parietal lobe, and the decision was made with our interdisciplinary epilepsy board to proceed with surgical resection.

#### VR-Assisted Preoperative Workup

2D DTI showed that the lesion was deep to the left descending corticospinal tract. Three-dimensional analysis further depicted the curvature of the precentral gyrus for the deep-seated pathology and provided a clearer form and shape to the diffuse lesion (Figure 3B). “Bottom-up” inspection using the Canopy approach of the relationship between the lesion and overlying motor fibers from within the depth of the operative corridor easily identified a safe and direct surgical corridor (Figure 3C and Video 3). 3D reconstruction of the patient's cortical and soft tissue anatomy in relation to the entry and target points guided head positioning to optimize corridor access and surgical ergonomics (Figure 3D and 3E).

#### Postoperative Course

Surgery was uncomplicated. Postoperatively, the patient had loss of sensation and proprioception in the left foot, which resolved in 3 months. Pathological diagnosis was FCD type 2A. He remained seizure-free at 2 years post-treatment with successful wean of all antiepileptic medications.

### Case 4

#### Presentation and Initial Management

An 11-year-old boy with a 9-year history of intractable gelastic seizures associated with a hypothalamic hamartoma is presented. He underwent 2 laser ablative procedures at age 2.5 years, after which his seizure type transformed to focal dyscognitive seizures and electrical status to epilepticus in sleep. Postablation scans showed residual hamartoma, and the decision was made with our interdisciplinary epilepsy board to proceed with additional ablative therapy.

#### VR-Assisted Preoperative Workup

**FIGURE 4.**
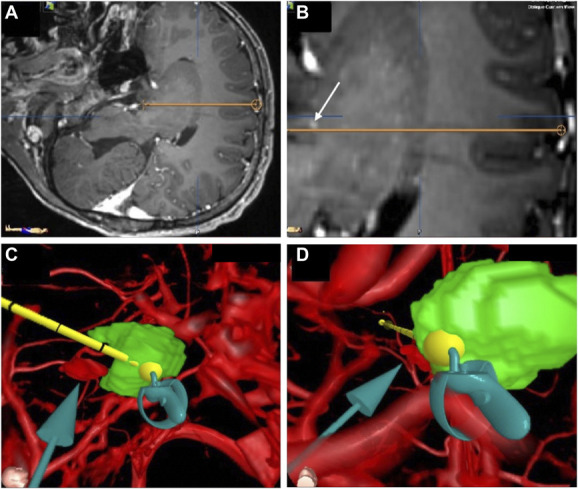
Trajectory planning for laser ablation of a hypothalamic hamartoma. **A**, Low-magnification trajectory view of the proposed laser fiber path. **B**, High magnification of the trajectory path reveals a potential contrast-enhancing vessel along trajectory. **C**, Side/intracranial and **D**, “Canopy” views of trajectory with on-the-fly, 3-dimensional adjustment of trajectory points.

Based on standard 2D imaging, linear contrast signal on T1 imaging was concerning for a traversing vessel. Attempts were made to design a single trajectory without threatening the vessel or missing the target (Figure 4A and 4B). VR modeling of the preliminary trajectory again suggested proximity to a structure that resembled a blood vessel. The “bottom-up” Canopy view proved to be most useful for identification of 2 trajectories for complete coverage of the lesion. Immersive navigation allowed further adjustment of the 3D points to ensure adequate clearance of the traversing vessel and suitable target points (Figure 4C and 4D and Video 4). Intraoperative magnetic resonance (MR) imaging showed no evidence of hemorrhage and satisfactory ablation of his residual hamartoma.

#### Postoperative Course

Surgery was uncomplicated, and the patient remained at their neurological baseline postoperatively. He remained seizure-free at 2 years post-treatment, at which time he was weaning off antiepileptic treatment.

### Case 5

#### Presentation and Initial Management

A 7-year-old boy with a 5-year history of intractable left temporoparietal focal epilepsy despite electrocorticography-guided resection of left anterior temporal focal cortical dysplasia Type 2A is presented. Postresection MRI did not show any residual imaging abnormality, and EEG from scalp recordings poorly localized seizure onset to diffuse left frontotemporal regions, thus necessitating sEEG. VR was used for planning sEEG electrode placement trajectories and subsequent laser ablation of seizure generating regions.

#### VR-Assisted Preoperative Workup

**FIGURE 5.**
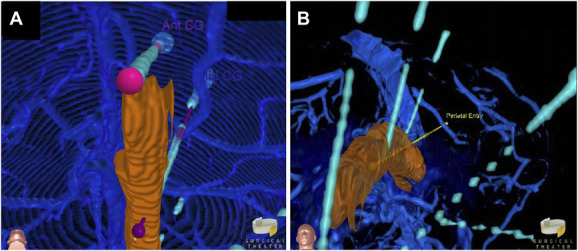
Stereo-EEG electrode placement and trajectory planning for laser ablation of the cingulate gyrus ictal onset zone. **A**, “Canopy” views of sEEG electrodes placed in the anterior (Ant CG) and middle (Mid CG) gyrus. Preplanned trajectories are blended with implanted sEEG electrodes. **B**, “Canopy” view of a laser ablation fiber implanted to a middle cingulate gyrus ictal onset zone through a parietal entry point. CG, cingulate gyrus; sEEG, stereo-electroencephalogram.

Preliminary sEEG trajectories were first proposed with standard 2D MR imaging and neuronavigation software and refined using VR and the “Canopy Approach,” which included electrodes into the anterior and middle cingulate gyrus (Figure 5A). sEEG monitoring revealed a middle cingulate gyrus seizure onset as a target for laser ablation. 2D planning resulted in a proposal for focal ablation of the middle cingulate along the previous sEEG tract. However, because of lack of well-defined anterior and posterior boundaries, the “canopy approach” was used to plan delivery of laser ablation along a length of the middle cingulate centered around the electrode target, which favored a path through the core of the cingulate gyrus extending through the superior parietal gyrus (Figure 5B and Video 5).

#### Postoperative Course

Laser ablation was performed without complication. The patient remained seizure-free for 18 months with significant improvement in language function and behavior. During the second postoperative year, seizures recurred, albeit with decreased frequency than before, with new posterior parietal foci noted on ictal single-photon emission computed tomography (SPECT). He is currently undergoing continued workup for seizure management.

## DISCUSSION

VR has emerged as a technology that can help neurosurgeons preoperatively identify ideal surgical approaches and important patient-specific anatomic landmarks to minimize intraoperative risk.^3,5,9,14,18^ Our demonstrative cases describe advantages of explicitly using a “bottom-up” Canopy approach for trajectory planning in a variety of neurosurgical cases. The canopy approach may also be useful to gauging the width of different trajectory corridors, which may be useful in cases where retraction would be necessary to access deeper lesions or placement of low-temperature limits for laser ablation. In addition, the ability to view adjacent structures surrounding a lesion from within the target can provide orienting landmarks during surgery. Although it is likely that most users will view the lesion from multiple perspectives during surgical planning, we have found that systematic fly-throughs of operative trajectories from both “top-down” and “bottom-up” perspectives resulted in the best optimization of the final trajectory.

### Limitations

Limitations of this study include those inherent to a small case series. We did not have any quantitative measures of increased accuracy or comparison group to demonstrate improved outcomes using our approach. Because of the heterogeneity of cases, it was not possible to identify matched controls for a single-center cohort. Future prospective studies to evaluate the added advantage of this approach can be designed such that multiple surgeons will first plan a trajectory using 2D images only, then again using a “top-down” VR approach, and a third time using a “bottom-up” VR approach. Possible quantitative measures may include distance of trajectory and distance from major vessels and fiber tracks. A large randomized study would be required to demonstrate clinical advantages such as operating room time and outcomes such as seizure freedom and complications. A prospective study documenting patient and family experience before and after the VR experience will also provide stronger evidence for the utility of a wide adaptation of this technology in the clinic. Possible disadvantages of VR include cost of additional hardware, software, technical support and added preoperative planning time. Establishing a systematic stepwise approach for VR planning may optimize advantages of this capability while streamlining the process.

## CONCLUSION

We describe the utility of using a “bottom-up” Canopy approach for surgical planning using VR in a series of pediatric neurosurgical patients. Although less intuitive than the traditional surgeon view from a planned entry point to the lesion, imaging interface demonstrates substantial potential to elucidate complex spatial relationships between neurosurgical pathology and critical structures.

## Appendix

**VIDEO 1.** 3D virtual reality fly-through of hemorrhagic parietal synovial sarcoma abutting the sagittal sinus. A “top-down” perspective shows a lesion largely to the left of the sinus; however, a “bottom-up” view from the underside of the lesion reveals tumor along the underside of the sinus traversing to the contralateral side.

**VIDEO 2.** 3D virtual reality fly-through of neuroepithelial tumor of the young from a “top-down” perspective along the proposed posterior lateral trajectory avoiding the optic radiations. Navigation of the intracranial space surrounding the lesion reveals surrounding vessels, including an *en passage* vessel through the lesion.

**VIDEO 3.** 3D virtual reality fly-through of a focal region of cortical dysplasia from a “top-down” perspective along the proposed trajectory and “bottom-up” view of traversing anatomy to identify other potential trajectories for optimal planning.

**VIDEO 4.** 3D virtual reality fly-through of trajectory planning for laser ablation of a hypothalamic hamartoma first from a “top-down” perspective and then from a “bottom-up” view of surrounding anatomy and vascular to identify other potential trajectories.

**VIDEO 5.** 3D virtual reality fly-through of trajectory planning for laser ablation of the cingulate ictal onset zone with stereo EEG leads superimposed on model. An optimal trajectory was chosen from the perspective of a “bottom-up” view of overlying vasculature to design a trajectory along the long axis of the cingulate.
